# European Hedgehogs as Hosts for *Borrelia* spp., Germany

**DOI:** 10.3201/eid1306.070224

**Published:** 2007-06

**Authors:** Jasmin Skuballa, Rainer Oehme, Kathrin Hartelt, Trevor Petney, Thomas Bücher, Peter Kimmig, Horst Taraschewski

**Affiliations:** * Zoological Institute I, Karlsruhe, Germany; † Baden-Wuerttemberg State Health Office, Stuttgart, Germany

**Keywords:** Erinaceus europaeus, Borrelia spp, Germany, Borrelia spielmanii, hedgehog, letter

**To the Editor:** The European hedgehog, *Erinaceus europaeus*, is known to host a variety of tickborne pathogens, including the virus that causes tickborne encephalitis (*1*) and at least 3 species of the *Borrelia burgdorferi* sensu lato group: *B. burgdorferi* sensu stricto, *B. afzelii,* and *B. garinii* (*2*). Members of the *B. burgdorferi* s. l. group are the most common vectorborne pathogens of humans in central Europe (*3*). The role of hedgehogs as hosts for these pathogens is, therefore, of considerable epidemiologic interest. Hedgehogs are a common synanthropic species that live in urban, suburban, and rural environments (*4*) and are known to carry not only the hedgehog tick, *Ixodes hexagonus*, but also the most common European tick, *I. ricinus* (*2*,*5*). Both of these ticks are known vectors of *B. burgdorferi* s. l. and tickborne encephalitis virus; *I. ricinus* is the most important vector of both throughout Europe (*1*,*5*). To date, however, only limited information has been available on the role of the hedgehog as a host or reservoir for *B. burgdorferi* s. l. in Germany.

We report the presence of 3 species of the *B. burgdorferi* s. l. group in European hedgehogs from Germany. To our knowledge, this is the first report of these species in hedgehogs in this country and the first report of *B. spielmanii* (A14S) (*6*) from this host.

The investigated hedgehogs came from 2 sources: 9 from the ≈40 in an experimental plot in the city of Karlsruhe, state of Baden-Wuerttemberg, and the remainder from wild hedgehogs that had been brought to hedgehog care centers from various areas of Germany. All hedgehogs had died naturally, and tissue samples were taken from 43 animals (kidneys from 43, heart from 22, bladder from 33). The bodies had been frozen at –17°C before the samples were taken.

DNA isolation was done by using the Maxwell 16 Instrument and System (Promega, Madison, WI, USA). Tissue samples were 3×3×3 mm. To detect *B. burgdorferi* s. l., we used 2 PCR protocols. The first was a nested PCR done according to the method of Rijpkema et al. (*7*). The target for the PCR was the spacer region between 5S and 23S rRNA genes of *B. burgdorferi* s. l. The nested primers generated a product of 226 bp. The amplified products were analyzed by agarose gel electrophoresis. The second protocol, a LightCycler-PCR hybridization assay (Roche Diagnostics, Mannheim, Germany) (*8*), simultaneously detects and genotypes the 3 genomic groups of *B. burgdorferi* s. l. This assay was specific for *B. burgdorferi* senso stricto, *B. garinii*, and *B. afzelii* (*8*) but also amplified *B. spielmanii* and *B. valaisiana*. The target for the PCR was the OspA gene.

The PCR products of both systems were sequenced. For DNA sequencing reaction, the fluorescence-labeled didesoxynucleotide technology (Applied Biosystems, Darmstadt, Germany) was used. The sequenced fragments were separated, and the data were collected with an ABI PRISM 310 Genetic Analyzer (Applied Biosystems). The obtained sequences were then analyzed and compared by using BLAST (www.ncbi.nlm.nih.gov/BLAST).

For 6 hedgehogs, *Borrelia* spp. could be clearly defined by using both gene sequences. Two additional animals had positive results, but sequencing was not possible because of either too little DNA or a mixed infection. *B. spielmanii* DNA was detected in the kidneys of 2 hedgehogs: 1 from Karlsruhe and 1 from 30 km west of this city in the German federal state of Rhineland-Palatinate. When sequences were compared by using BLAST, 4 BLAST sequences (AM055823, AM055822, DQ133518, AY 995900) showed 100% similarity with *B. spielmanii*. *B. garinii* was detected in the heart of 2 animals (from Berlin and Karlsruhe); *B. afzelii* in 3 animals (in the kidney of 2 from Hamburg and Karlsruhe and in the bladder of 1 from Rhineland-Palatinate). A single animal (from Karlsruhe) had *B. afzelii* in the kidney and bladder and *B. garinii* in the heart. Preliminary results have also shown that ticks collected from hedgehogs from the Karlsruhe site were infected with *B. afzelii* (an *I. hexagonus* nymph and an *I. ricinus* female) and with *B. spielmanii* (an *I. ricinus* female, a nymph, and a larva) (Skuballa et al., unpub. data).

These results show, that hedgehogs harbor at least 3 of the 5 recognized *Borrelia* genospecies found in Germany, all of which are known (*B. afzelii*, *B. garinii*) or are strongly suspected (*B. spielmanii*) of being pathogens for humans (*9*,*10*). To our knowledge, ours is the first report of *B. spielmanii* from hedgehogs, a *Borrelia* sp. that is usually associated with rodents, especially with garden and hazel dormice (*10*). That *Borrelia* spp. infections commonly occur in European hedgehogs is likely. However, questions remain about the role of these pathogens in regulating the populations of European hedgehogs and about the status of these common synanthropic mammals as a reservoir host of *B. burgdorferi* s. l. in periurban and rural environments.
